# Cost-of-Illness and the Health-Related Quality of Life of Patients in the Dengue Fever Outbreak in Hanoi in 2017

**DOI:** 10.3390/ijerph15061174

**Published:** 2018-06-05

**Authors:** Bach Xuan Tran, Giang Thu Vu, Long Hoang Nguyen, Anh Tuan Le Nguyen, Tung Thanh Tran, Binh Thanh Nguyen, Thao Phuong Thi Thai, Carl A. Latkin, Cyrus S. H. Ho, Roger C. M. Ho

**Affiliations:** 1 Institute for Preventive Medicine and Public Health, Hanoi Medical University, Hanoi 100000, Vietnam; dennguyenle@gmail.com; 2 Bloomberg School of Public Health, Johns Hopkins University, Baltimore, MD 21205, USA; carl.latkin@jhu.edu; 3 Vietnam Young Physician Association, Hanoi 100000, Vietnam; 4 Institute for Global Health Innovations, Duy Tan University, Da Nang 550000, Vietnam; gigi.vugiang@gmail.com (G.T.V.); tung.ighi@gmail.com (T.T.T.); binh.ighi@gmail.com (B.T.N.); 5 School of Medicine and Pharmacy, Vietnam National University, Hanoi 100000, Vietnam; longnh.ph@gmail.com; 6 Department of General Planning and Department of Cardiology, Friendship Hospital, Hanoi 100000, Vietnam; thaithaohp2014@gmail.com; 7 Department of Psychological Medicine, National University Hospital, Singapore 119074, Singapore; su_hui_ho@nuhs.edu.sg; 8 Department of Psychological Medicine, Yong Loo Lin School of Medicine, National University of Singapore, Singapore 119228, Singapore; pcmrhcm@nus.edu.sg

**Keywords:** dengue fever, quality of life, cost of illness, Vietnam

## Abstract

Dengue fever (DF) outbreaks occur intermittently in Vietnam, and the most recent epidemic happened in 2017. However, attempts to measure the burden of DF in relation to the quality of life and the cost of treatment for patients during an epidemic period are constrained. This study explored the health-related quality of life (HRQOL) and the cost of illness among patients with dengue fever in Vietnam. A cross-sectional study was conducted in Bach Mai Hospital from September to November 2017. The EuroQol-5 dimensions-5 levels (EQ-5D-5L) was used to measure HRQOL. Cost-of illness was measured by collecting data about the direct medical cost, the non-medical cost, and the indirect cost. Among 225 patients, most of the participants experienced problems regarding mobility (62.3%), self-care (71.8%), usual activities (64.6%), and anxiety/depression (64.1%). The mean EQ-5D index was 0.66 (SD = 0.24). The median cost of illness for inpatient and outpatient groups were US $110.10 (IQR = US $4.40–1200.00) and US $36.10 (IQR = US $1.80–816.30), respectively. Indirect costs accounted for a major proportion in both groups. Lower-skilled workers and those with a higher severity of the disease had significantly lower HRQOL. Meanwhile, people who were inpatients, had comorbidities, had higher incomes, and who experienced a longer disease duration, had a higher cost of treatment. In conclusion, high costs and severe health deterioration, especially in psychological dimensions, were found in patients with DF in Vietnam. Strengthening primary health care services and communication campaigns are necessary to relieve the burden of diseases and could possibly contribute to effective DF control and prevention strategies.

## 1. Introduction

Dengue is one of the most prevalent mosquito-borne viral infections, and has been a global health problem for many decades [1]. A global estimate has shown a 7-time increase of the incidence of dengue from 1990 (8.3 million cases) to 2013 (58.4 million cases), particularly in tropical and subtropical regions, such as Latin America, South-East Asia, and the Western Pacific [2]. No specific treatment has been found for the disease, while prevention and control efforts have achieved limited success, especially in developing areas [3,4].

Several attempts have been made to quantify the burden of dengue [2,5,6,7]. Alongside morbidity and mortality, quality of life (QOL) is a critical metric for measuring the health burden of chronic and acute illnesses. In literature, dengue fever (DF) substantially deteriorates patients’ QOL [8,9]. In Brazil, Martelli et al. indicated that patients with DF had a poor QOL, especially in terms of cognition, self-care, and pain [10]. Meanwhile, in Malaysia, Lum LC et al. found that patients were mostly affected in terms of their cognition and interpersonal activities [11]. Age, duration of illness, and the severity of the disease were found to be associated with the QOL of patients with DF [5,10,11]. Furthermore, DF causes a significant economic burden, due to its high cost of treatment. Lee et al. (2017) reported the total cost per DF episode to be in the range of US $141–385 for hospitalized patients, and US $40–158 for non-hospitalized patients in Vietnam, Thailand, and Colombia [6]. The economic cost of DF was found to be 0.03% of the GDP per capita in South East Asia in 2010 [7]. The indirect costs or the costs caused by productivity loss due to DF have been cited by several studies as a main source of costs [6,7]. Since the data about health-related quality of life (HRQOL) and the cost of treatment is limited and varied across settings, it is vital to provide more evidence to estimate the negative impacts of DF.

In Vietnam, DF is common and widespread, with cases of infection being reported year-round. The country has experienced two DF outbreaks in the last decade. The first one happened in 2009, with around 235 thousand reported incidents, of which 377 were fatal. In 2017, the outbreak occurred from July to December, with 184 thousand people infected, including 32 deaths [12,13]. These statistics were 1.5 times higher compared to the number of cases (122 thousand people) in 2016 [13]. The epidemic was mostly concentrated in urban areas, such as Hanoi and Ho Chi Minh city, resulting in an overload on the hospital system in Vietnam, particularly in central hospitals with infectious departments. Albeit a prevalent disease, only limited studies have explored the effects of DF on the quality of life of patients, as well as the financial burden that is suffered by those who are infected in Vietnam [14,15,16]. This data is critical in supporting the decision-making processes of clinical practitioners and will partly contribute to further economic evaluations for DF prevention, cure, and control interventions. Therefore, this study aims to examine the HRQOL and the cost of illness among patients with dengue fever in Vietnam, as well as to explore the potential associated factors.

## 2. Materials and Methods 

### 2.1. Study Site

A cross-sectional study was conducted in the Infectious Diseases Department (IDD) of Bach Mai Hospital, from September to November 2017. Bach Mai hospital is the leading central hospital in Northern Vietnam [17]. Since the beginning of July 2017, the IDD has received an average of 50 to 70 DF patients per day. In August 2017, the department received and treated 1189 DF patients [18]. 

The participants were included in the study if they met five eligibility criteria as follows: (1) Visiting the IDD, irrespective of whether they were an inpatient or outpatient; (2) Having DF or symptoms of DF; (3) Having a confirmed positive result with the NS1 Ag/IgM/IgG rapid test for the dengue virus; (4) Agreeing to participate in this interview; and (5) Having the ability to answer the questionnaire in 10–15 min. We used a convenience sampling method to recruit the patients. A total of 223 patients enrolled in the study.

### 2.2. Measurements and Instruments

A structured questionnaire was developed and was used for face-to-face interviews with the patients. Each interview lasted 10–15 min. The eligible patients had been introduced to the study and they provided written informed consent if they agreed to participate. We invited the participants to a private area of the clinic to ensure confidentiality and the quality of the interview. The collected information is described below:

#### 2.2.1. Social-Demographic and Epidemiological Characteristics 

We collected data about age, gender, education, marital status, employment, and living area. Information including the severity of dengue fever, the duration of the disease, and whether patients had comorbidities was also collected.

#### 2.2.2. Health-Related Quality of Life (HRQOL)

The EuroQol-5 dimensions-5 levels (EQ-5D-5L) instrument was employed to measure the HRQOL in five dimensions: Mobility, Self–Care, Usual Activities, Pain/Discomfort, and Anxiety/Depression. Each of these dimensions had five levels for the responses, from no problem to extremely problematic [19], resulting in 3125 health states. By using a cross-walk value set of the Thailand population, 3125 single EQ-5D indexes could be produced from these health states.

#### 2.2.3. Cost of Dengue Fever Treatment

Costs per inpatient and outpatient visit were computed by asking the patients to report their expenditure due to DF. Data collectors helped the patients to list all of the cost components for DF treatment. The patients then estimated the costs by each activity. The unit costs comprised of three elements: (1) medical expenditure (drugs, lab tests, hospital fees, and other); (2) non-medical spending (transportation, accommodation, and special meals, if any); and (3) Indirect costs (loss of revenue due to absence from work for both patients and caregivers).

### 2.3. Statistical Analysis

Data analysis was performed using Stata version 12.0 (Stata Corp. LP College Station United States of America, Lakeway, TX, USA). Because the data on the cost of treatment were not normally distributed, we transformed this data into the log form, which allowed us to conduct the regression model. Multivariate Tobit and Linear regression models were applied to determine the factors that were associated with the patients’ HRQOL and the cost of treatment, respectively. Stepwise forward strategies were used, along with regression models, to construct reduced models, and a *p*-value of < 0.2 of the log-likelihood ratio test was used for the variable selections.

### 2.4. Ethical Approval

The study protocol was approved by the Institutional Review Board (IRB) of Hanoi Medical University (Code: 03.18/HDDDDYHN). Before being interviewed, the patients were asked to give written informed consent if they agreed to participate. The participants could withdraw from the study at any time without this having any effect on their current treatment. Their data was kept in safe places, and only the principal investigators could access the data. 

## 3. Results

Of the 223 patients interviewed, the mean age was 31.6 (SD = 12.4) years, 48.9% were female, 80.7% had completed high school education or above, 52.0% were married or living with a partner, 72.6% were freelancers or were under some form of employment, and 81.2% were urban citizens. Reportedly, 64.6% were not covered by health insurance. The poorest income quintiles accounted for 39.9% of the respondents, while the figure for the richest income quintile was 19.6%. Statistically significant differences in participant characteristics between inpatient and outpatient services were found for marital status, living location, and age (*p* < 0.05) (Table 1).

The majority of respondents reported having mild DF (55.6%) and no comorbidities (68.6%). The duration of the disease was shorter for the outpatient group (with an average of 4.9 days versus 5.9 days for the inpatient group). As for HRQOL, the majority of participants claimed to experience problems in terms of mobility (62.3%), self-care (71.8%), usual activities (64.6%), and anxiety/depression (64.1%), while a minority reported experiencing pain/discomfort (32.2%). Overall, the EQ-5D index was 0.66 (SD = 0.25), and no difference was found between inpatients and outpatients (Table 2). 

Table 3 shows that the median total of the non-medical cost for the inpatient group was US $19.80 (US $1.80–242.30), versus US $8.80 (US $0.70–220.30) for the outpatient group, of which food accounted for a large proportion. The median total of the direct medical cost for the hospitalized patients was US $132.20 (US $26.40–308.40), of which user fee was a major component—4.8 times more than that of non-hospitalized ones. The median indirect cost for the inpatient and outpatient service types were US $88.10 and US $44.10, however inpatient service had a much wider cost range (US $4.40–1189.40, versus US $8.80–118.90).

Table 4 presents the factors that were associated with HRQOL and the cost of DF treatment. Being a blue-collar worker/farmer and having a higher level of disease severity were found to be significantly correlated with lower HRQOL. Meanwhile, people who had a college/vocational training degree had a higher HRQOL score compared to those who had less than a high school education (Coef. = 0.18; 95% CI = 0.03–0.34). The cost of treatment for inpatients was found to be significantly higher than for outpatients. People who were in the richer quintiles paid significantly more than those of lower income groups. Notably, a higher duration of disease was positively associated with a higher cost of treatment.

## 4. Discussion

This study provided evidence that substantially reduced HRQOL is associated with DF among Vietnamese patients. Moreover, the patients not only suffered from decreased physical health, but they also suffered severe psychological health problems. We also observed a significantly high cost of treatment for these patients, particularly for those who were hospitalized. Indirect medical cost, which were measured using productivity loss, remained as the major component, when direct costs accounted for one third of the total cost of the illness. These findings highlight the necessity of strengthening the local health facilities for managing dengue patients, and implementing educational campaigns about appropriate health care and counselling for the patients.

Compared to previous studies, participants in this study were older (33.9 ± 14.3) (the reported average age of patients in studies conducted nationally in 2011 and at Cu Chi General Hospital in 2016, was 24 ± 7.2 years and 23.2 ± 13.3 years, respectively) [14,15]. This indicated that working adults are still most vulnerable to the virus, albeit more so in the higher age group. People who were younger, single, better educated, and living in urban areas appeared to favor non-hospitalized treatment. Meanwhile, about one third of inpatients came from rural areas. Possible reasons for this might be the fact that many patients from rural areas had delayed access to health services and were therefore transferred to central hospitals because of the severity of their health status. In addition, some patients from the inner city of Hanoi had the belief that hospitalization was not necessary to cure DF, or mentioned the rather obvious fact that hospitalization incurred a much higher cost [3,15]. 

In this study, HRQOL of our patients (EQ-5D index) was only equal to two thirds of HRQOL of the general Vietnamese population, suggesting a clinically significant reduction of HRQOL in patients with DF [20]. In addition, comparable to the finding of the general population study, no significant correlation was found between the age and gender of dengue patients and their HRQOL [20]. This may indicate the similarities of the vulnerabilities to dengue and the ease of access to treatment services for male and female Vietnamese people from different age groups. Thus, a universal dengue treatment policy can be applied, regardless of the age and gender of who is infected. Alongside problems in self-care, usual activities, and mobility, we found that two thirds of patients (both inpatients and outpatients) suffered from anxiety/depression. This is partly because of the regular media coverage regarding severe dengue cases and the deaths during the period of this study which triggered worries for the patients about their health until they went to hospital. 

The median cost of treatment in this study was considerably higher compared to a similar study, which reported a mean total direct medical cost, a direct non-medical cost, and an indirect cost of US $47.10 ± 31.9, US $41.10 ± 38, and US $51 ± 22.70, respectively [15]. This may be due to the lack of health insurance that was utilized among the patients that were interviewed. The significantly higher treatment cost that was experienced by inpatients compared to non-hospitalized ones may reflect the fact that patients tend to prefer using health services at a higher level of administration, resulting in a high cost for transportation and the loss of productivity for caregivers. Hence, efforts should be made to familiarize citizens with the use of health insurance, encouraging local health service use, especially for those from rural areas. On the other hand, the significantly higher cost of treatment incurred for people in the richest income quintiles may just reflect their ability, and thus their willingness, to pay more compared to the poorer groups, indicating the potential to introduce home-based care with extra payments. 

This study has implications for clinical practice, as well as for policy and management. As for the clinical aspect, patients with DF had a significant deterioration in health utility, especially regarding the psychological health dimension. Therefore, appropriate counseling via phone or family doctors, and early access to health services should be promoted, especially during the peak season of the epidemics. In addition, when communicating about dengue control and prevention, the media should also encourage patients to seek out and use local health service providers in order to reduce the heavy loads at central levels, as well as the high costs that are associated with the illness. Lastly, dengue has remained a high burden epidemic in Vietnam—both from economic and health perspectives. Therefore, effective policies are needed to prevent and manage it, involving the community, family, and the health system.

This study also has some limitations. Since it surveyed patients at central hospitals, many of them were more severe and therefore, may not be representative of the whole population. In addition, the patients might come from diverse areas with different patterns of service use prior to being admitted to hospital. Moreover, we could not include people with mild dengue who did not visit the hospital for treatment, who might be one of the most vulnerable populations. Therefore, further studies of this group should be warranted in the future. Besides, as a cross-sectional study, we asked the patients to recall their expenditure, which might lead to bias in measuring costs. Nonetheless, as evidence for this topic is still scarce, the findings of this study may contribute to the improvement of dengue control and to prevention programs in Vietnam.

## 5. Conclusions

In conclusion, a high cost and severe health deterioration, especially in the psychological dimension, were associated with dengue among patients in Vietnam. Strengthening local health services, improving the coverage and content of communication campaigns, and setting counselling channels are necessary to relieve the burden of diseases, and may also contribute to the effectiveness of dengue control and to its prevention in Vietnam.

## Figures and Tables

**Table 1:** Demographic characteristics of the respondents.

Characteristics	Inpatient		Outpatient		Total		*p*-Value
	*n*	%	*n*	%	*n*	%	
Total	105	47.1	118	52.9	223	100.0	
Gender							
Female	47	44.8	62	52.5	109	48.9	0.25
Male	58	55.2	56	47.5	114	51.1	
Education attainment							
Secondary school or below	28	26.7	15	12.7	43	19.3	0.06
High school	28	26.7	35	29.7	63	29.3	
Vocational training/College	11	10.5	13	11.0	24	10.8	
University	38	36.1	55	46.6	93	41.7	
Marital status							
Single	39	37.1	64	54.2	103	46.2	0.04
Living with spouse/partner	64	61.0	52	44.1	116	52.0	
Divorce/Widow	2	1.9	2	1.7	4	1.8	
Occupations							
Unemployed	9	8.6	5	4.2	14	6.3	0.34
Freelancers	30	28.6	30	25.4	60	26.9	
White-collar workers	22	21.0	30	25.4	52	23.3	
Blue-collar workers/Farmers	13	12.4	8	6.8	21	9.4	
Students	18	17.0	29	24.6	47	21.1	
Others	13	12.4	16	13.6	29	13.0	
Living location							
Urban	76	81.2	105	89.0	181	81.2	<0.01
Rural	29	27.6	13	11.0	42	18.3	
Having health insurance							
Yes	34	32.4	45	38.1	79	35.4	0.37
No	71	67.6	73	61.9	144	64.6	
Household income quintiles							
Poorest	24	34.3	31	45.6	55	39.9	0.48
Poor	1	1.4	0	0.0	1	0.7	
Middle	15	21.4	12	17.7	27	19.6	
Rich	17	24.3	11	16.2	28	20.3	
Richest	13	18.6	14	20.6	27	19.6	
	**Mean**	**SD**	**Mean**	**SD**	**Mean**	**SD**	
Age	33.9	14.3	29.4	10.0	31.6	12.4	<0.01

**Table 2:** Health status and the health-related quality of life of patients with dengue fever.

Characteristics	Inpatient		Outpatient		Total		*p*-Value
	*n*	%	*n*	%	*n*	%	
Having problems with mobility							
Yes	66	62.9	73	61.9	139	62.3	0.88
No	39	37.1	45	38.1	84	37.7	
Having problems with self-care							
Yes	71	67.6	89	75.4	160	71.8	0.20
No	34	32.4	29	24.6	63	28.3	
Having problems with usual activities							
Yes	67	63.8	77	65.3	144	64.6	0.82
No	38	36.2	41	34.8	79	35.4	
Pain/Discomfort							
Yes	37	35.2	35	29.7	72	32.3	0.37
No	68	64.8	83	70.3	151	67.7	
Anxiety/Depression							
Yes	67	63.8	76	64.4	143	64.1	0.93
No	38	36.2	42	35.6	80	35.9	
Severity of dengue fever							
Mild	53	52.5	66	58.4	119	55.6	<0.01
Symptomatic	16	15.8	33	29.2	49	22.9	
Severe	32	31.7	14	12.4	46	21.5	
Having comorbidities							
Yes	39	37.1	31	26.3	70	31.4	0.08
No	66	62.9	87	73.7	153	68.6	
	**Mean**	**SD**	**Mean**	**SD**	**Mean**	**SD**	
EQ-5D index	0.66	0.25	0.66	0.23	0.66	0.24	0.99
Duration of disease (days)	5.9	2.4	4.9	2.9	5.4	2.7	<0.01

**Table 3:** Cost of treatment among patients with dengue fever (Unit: USD).

Characteristics	Inpatient				Outpatient			
	*n* ^†^	Median	IQR [p25–p75]		*n* ^†^	Median	IQR [p25–p75]	
Direct non-medical cost								
Food	46	12.8	2.2	66.1	16	19.8	8.8	88.1
Travel	62	13.2	0.9	220.3	87	8.8	0.7	110.1
Total non-medical cost	73	19.8	1.8	242.3	92	8.8	0.7	220.3
Direct medical cost								
Drug	3	88.1	0.6	440.5	14	28.6	13.2	44.1
Test	10	26.8	26.4	35.2	35	22.0	4.4	88.1
User fee (or bed)	13	189.4	132.2	220.3	1	30.8	-	-
Other	3	132.2	13.2	176.2	7	8.8	8.8	8.8
Total medical cost	22	132.2	26.4	308.4	41	27.3	4.4	132.2
Indirect cost								
Patients	55	74.0	10.6	1189.4	58	38.3	8.8	308.4
Caregivers	38	57.3	4.4	508.8	32	26.4	5.7	118.9
Total indirect cost	73	88.1	4.4	1189.4	67	44.1	8.8	495.6
Total	105	110.1	4.4	1200.0	118	36.1	1.8	816.3

^†^*n*: Number of patients who responded to the questions; IQR: interquartile range.

**Table 4:** Factors associated with quality of life and the transformed logarithm of the cost of dengue fever treatment among respondents.

	EQ-5D Index		Cost of Treatment	
	Coef.	95% CI	Coef.	95% CI
Type of patients (Inpatient vs Outpatient)	−0.01	−0.11; 0.08	0.64 **	0.10; 1.18
Education attainment (vs < High school)				
College/Vocational training	0.18 **	0.03; 0.34		
Occupation (vs Unemployed)				
Blue-collar worker/Farmer	−0.24 ***	−0.40; −0.09	0.97 **	0.11; 1.82
Other	−0.20 ***	−0.34; −0.06	0.66 *	−0.10; 1.42
Having health insurance (Yes vs No)	−0.08 *	−0.18; 0.01		
Income quintiles (vs Poorest)				
Rich			0.56 *	−0.10; 1.22
Richest			0.90 ***	0.23; 1.57
Severity of disease (vs Mild)				
Symptomatic	−0.13 **	−0.25; −0.02		
Severe	−0.16 **	−0.28; −0.03		
Duration of disease (days)	0.02	−0.00; 0.03	0.16 ***	0.05; 0.27
Having comorbidity (Yes vs No)			0.44	−0.15; 1.03

* *p* < 0.1; ** *p* < 0.05; *** *p* < 0.01; 95% CI: 95% Confident interval, Coef.: Coefficient.
